# Supplementary material for: Role of Klotho and AGE/RAGE-Wnt/β-Catenin Signalling Pathway on the Development of Cardiac and Renal Fibrosis in Diabetes
Source: Int J Mol Sci. 2023 Mar 9;24(6):5241. doi: 10.3390/ijms24065241 (PMC10049403; doi:10.3390/ijms24065241)
Supplement: Supplementary file 1 [file ijms-24-05241-s001.zip › Figure S1.pdf]

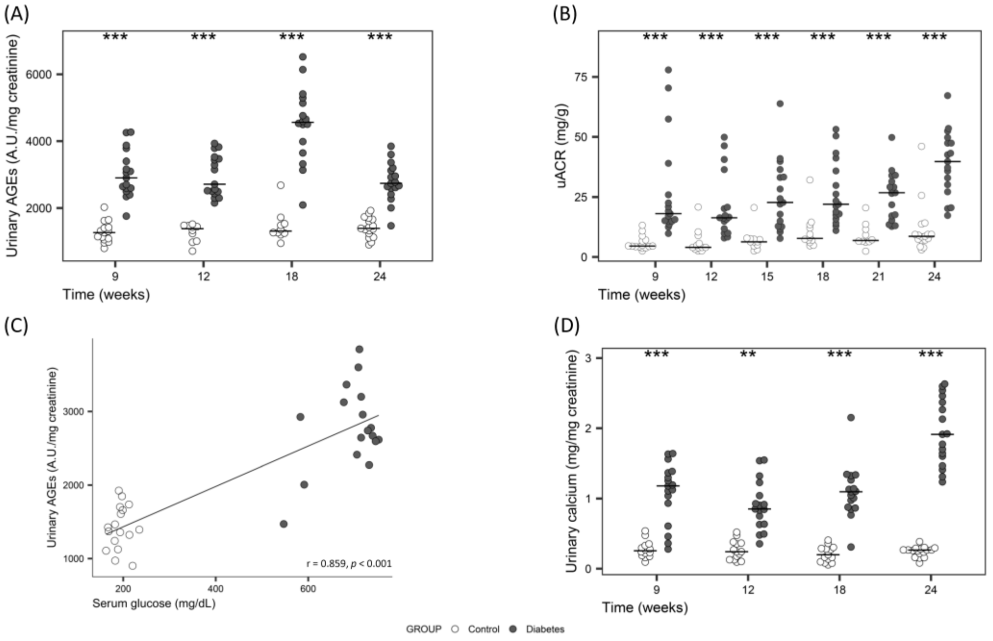

Figure S1. Time course of (**A**) urinary AGEs and (**B**) uACR in controls and diabetic rats. (**C**) Correlation between urinary AGEs and serum glucose adjustment for diabetes. (**D**)Time course of urinary calcium in controls and diabetic rats. uACR: urinary albumin/creatinine ratio; AGEs: advanced glycation end products. Non-parametric Wilcoxon rank sum test was used. Horizontal line represents median. ** $p \leq 0.01$ vs. control, *** $p < 0.001$ vs. control.
