# Supplementary material for: Role of Klotho and AGE/RAGE-Wnt/β-Catenin Signalling Pathway on the Development of Cardiac and Renal Fibrosis in Diabetes
Source: Int J Mol Sci. 2023 Mar 9;24(6):5241. doi: 10.3390/ijms24065241 (PMC10049403; doi:10.3390/ijms24065241)
Supplement: Supplementary file 1 [file ijms-24-05241-s001.zip › Figure S2.pdf]

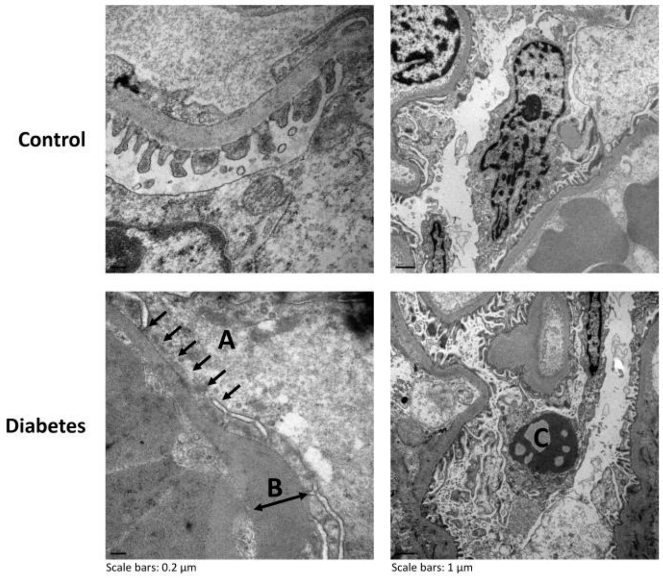

Figure S2. Electron microscope representative images of renal ultrastructure in control and diabetic rats. (**A**) Indicates podocyte foot process effacement. (**B**) Indicates diffuse thickening basement membrane. (**C**) Podocyte pyknotic nuclei.
