# Supplementary material for: Role of Klotho and AGE/RAGE-Wnt/β-Catenin Signalling Pathway on the Development of Cardiac and Renal Fibrosis in Diabetes
Source: Int J Mol Sci. 2023 Mar 9;24(6):5241. doi: 10.3390/ijms24065241 (PMC10049403; doi:10.3390/ijms24065241)
Supplement: Supplementary file 1 [file ijms-24-05241-s001.zip › Figure S4.pdf]

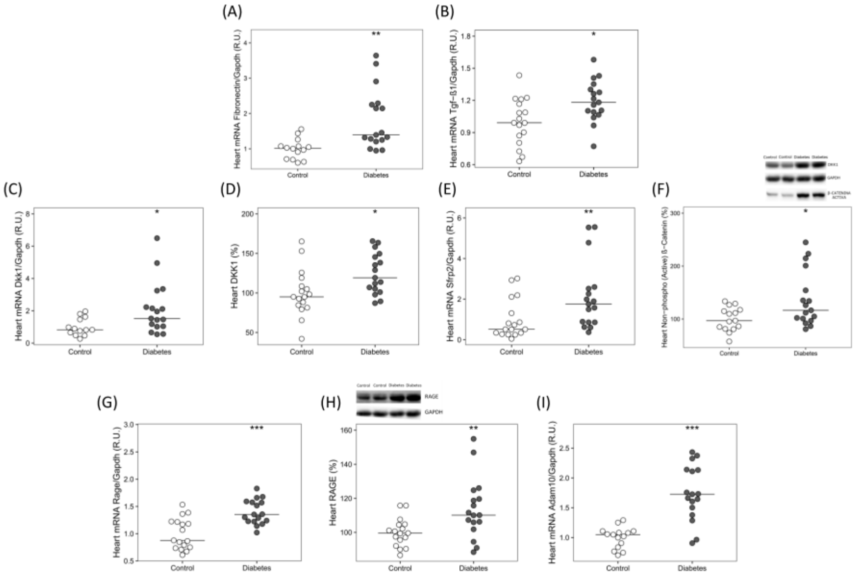

Figure S4. Heart gene expression of (**A**) Fibronectin; and (**B**) Tgf-β1. Wnt pathway markers: (**C**) Gene expression of Dkk1; and (**D**) Relative quantification and representative image of Western blot analysis of DKK1. (**E**) Gene expression of Sfrp2. (**F**) Relative quantification of Western blot analysis and representative image active ß-catenin. (**G**) Gene expression of Rage; (**H**) Relative quantification and representative image of Western blot analysis of RAGE; and (**I**) Gene expression of Adam10. R.U.: relative units. Non-parametric Wilcoxon rank sum test was used. Horizontal line represents median. * $p \leq 0.05$ vs. control, ** $p \leq 0.01$ vs. control, *** $p \leq 0.001$ vs. control.
